# MWCNT Coated Free-Standing Carbon Fiber Fabric for Enhanced Performance in EMI Shielding with a Higher Absolute EMI SE

**DOI:** 10.3390/ma10121350

**Published:** 2017-11-24

**Authors:** Sudesh Jayashantha Pothupitiya Gamage, Kihun Yang, Ramanaskanda Braveenth, Kanthasamy Raagulan, Hyun Suk Kim, Yun Seon Lee, Cheol-Min Yang, Jai Jung Moon, Kyu Yun Chai

**Affiliations:** 1 Division of Bio-Nanochemistry, College of Natural Sciences, Wonkwang University, Iksan City 570-749, Korea; jayashanthapgs@gmail.com (S.J.P.G.); kihun0432@wku.ac.kr (K.Y.); braveenth.czbt@gmail.com (R.B.); raagulan@live.com (K.R.); 2 RIC for Next Generation Industrial Radiation Technology, College of Natural Sciences, Wonkwang University, Iksan City 570-749, Korea; nanohskim@wku.ac.kr; 3 Multifunctional Structural Composite Research Center, Institute of Advanced Composite Materials, Korea Institute of Science and Technology, Chudong-ro 92, Bongdong-eup, Wanju-gun, Jeollabukdo 55324, Korea; t14225@kist.re.kr; 4 Clean & Science Co., Ltd., Jeongeup 3 Industrial Complex 15BL, 67, 3sandan 3-gil, 56136 Buk-myeon, Jeongeup-si, Korea; jjmoon@cands.kr

**Keywords:** MWCNT, carbon fiber, fabric, EMI shielding, absolute EMI SE

## Abstract

A series of multi-walled carbon nanotube (MWCNT) coated carbon fabrics was fabricated using a facile dip coating process, and their performance in electrical conductivity, thermal stability, tensile strength, electromagnetic interference (EMI) and shielding effectiveness (SE) was investigated. A solution of MWCNT oxide and sodium dodecyl sulfate (SDS) in water was used in the coating process. MWCNTs were observed to coat the surfaces of carbon fibers and to fill the pores in the carbon fabric. Electrical conductivity of the composites was 16.42 S cm^−1^. An EMI shielding effectiveness of 37 dB at 2 GHz was achieved with a single layer of C/C composites, whereas the double layers resulted in 68 dB EMI SE at 2.7 GHz. Fabricated composites had a specific SE of 486.54 dB cm^3^ g^−1^ and an absolute SE of approximately 35,000 dB cm^2^ g^−1^. According to the above results, MWCNT coated C/C composites have the potential to be used in advanced shielding applications such as aerospace and auto mobile electronic devices.

## 1. Introduction

Electromagnetic interference (EMI) is a radiated electromagnetic signal-intrusion generated by an external source which can affect and perturb the function of nearby electronic equipment such as circuits. EMI can arise from various sources and devices that distribute or process any form of electrical energy are a major source of EMI radiation [1]. The rapid development of modern electronic products which results in abundant use of electronic devices and highly integrated circuits, generate significant amounts of electromagnetic radiation, thus producing adverse effects on living beings as well as highly sensitive, precision electronic equipment. EMI radiation is especially harmful to human beings. When humans are exposed to electromagnetic (EM) radiation, it leads to heat buildup in sensitive tissues inside the body such as eyes and veins [2]. It has also been found to considerably increase the chromosome aberration frequency in micronuclei in white blood cells which may give rise to unwanted mutations [3].

EMI shielding is crucial to protect humans and circuits from EMI radiation. Focused attention has been given to achieve high-performance EMI shielding materials due to the increasing attention given by electronic and communication industries as a result of prevailing issues arising from EMI.

There are various types of shielding materials metal based fabrics, composites, carbon filler containing composites, etc. Composites with conducting filler materials such as metal flacks, carbon allotropes have been widely used to increase the EMI shielding effectiveness [4,5,6,7,8,9,10,11,12]. Conventional EMI shielding materials consist of various metals such as Cu and Ni [11,12]. These conductive materials have certain limitations. For example, aluminum based shields have low impact resistance, and stainless steel ones have high density. Metals are prone to corrosion, especially under conditions such as salinity. Interactions with corroded metals result in radio interference which is known as the Rusty Bolt Effect. Galvanic corrosion may result if two different metals are used in making the shield. Thus, the use of metals adds complexity to the manufacturing effective metal shields [12,13,14].

As a recent advent in the shield manufacturing, carbon materials have been used in the development of electrically conductive polymer composites, fabrics and textiles. This became a success mainly due to low cost, corrosion resistance, flexibility, and large aspect ratio of carbon materials [4,5,6,7,8,9,15]. The EMI shielding effectiveness of composites and fabrics depends on their intrinsic electrical conductivity, magnetic permeability and aspect ratio [7].

Carbon-based materials such as carbon fibers, multi-walled carbon nanotubes (MWCNT), single-walled carbon nanotubes (SWCNT), and chemically modified graphite and graphene (GN) have been used either as conductive fillers for composites or as base material for films because of their high electrical conductivity, light weight, flexibility, and large aspect ratio [4,5,6,7,8,9,15]. Polymer composites consist of individual carbon nanoparticles that are randomly distributed in the polymer matrix and surrounded by polymer chains. The electrical conductivity of these polymers strongly depends upon electron percolation between separated nanofillers [4,5,6,7,8,9]. It has been experimentally demonstrated that 7 wt % MWCNTs loading in polystyrene [16], 15% SWCNTs [15] or 15% graphene [7] is sufficient to achieve an industrial standard level of EMI shielding effectiveness 20 dB. However, increased loading of conductive filler material may lead to decreased mechanical properties and lower processability due to agglomeration [3].

Non-woven carbon fabrics are different compared to polymer based composites and can be identified as a carbon fiber reinforced matrix composites (C/C). The non-woven carbon fabrics have numerous applications such as being used in conductive anodes in lithium ion batteries [17] activated carbon fabrics for air absorption [18], glucose oxidase biosensors based on carbon nanotube non-woven fabric [19] and they can also be modified to be used in EMI shielding [20].

Carbon fiber reinforced matrix composites (C/C) are normally fabricated by chemical vapor infiltration and pyrolysis and this fabrication process leads to the inevitable formation of cracks in the carbon material. These cracks and nanopores could lead to decreased density of the material and may be detrimental to the electrical conductivity also leading to lower EMI shielding effectiveness. As CNTs are excellent EMI shielding material [4,6,8,20,21] and light weight microwave absorbers, they have been widely used in polymer-based EMI shielding composites. It was experimentally proved that the EMI shielding effectiveness of CNT reinforced the C/C (Carbon/Carbon) composites [20] to increase the EMI shielding effectiveness of C/C materials compensating the adverse effects of cracks and nanopores, coating CNTs into annular gaps and pores in the C/C composites seems to be a highly promising method.

In the current study, non-woven carbon fabric coated with MWCNT was developed to produce a composite with high EMI shielding. Non-woven carbon cloth fabric was coated with MWCNT to obtain enhanced EMI shielding effectiveness. The content of the coating was adjusted using dipping and drying cycles. The effect of MWCNT amount on the microstructure, chemical structure, percentages of elements, tensile strength, electrical conductivity, and EMI shielding effectiveness were investigated in detail.

## 2. Results and Discussion

### 2.1. Structural Characterization

#### 2.1.1. Scan Electron Microscopic Analysis of Morphology

**Figure 1:**
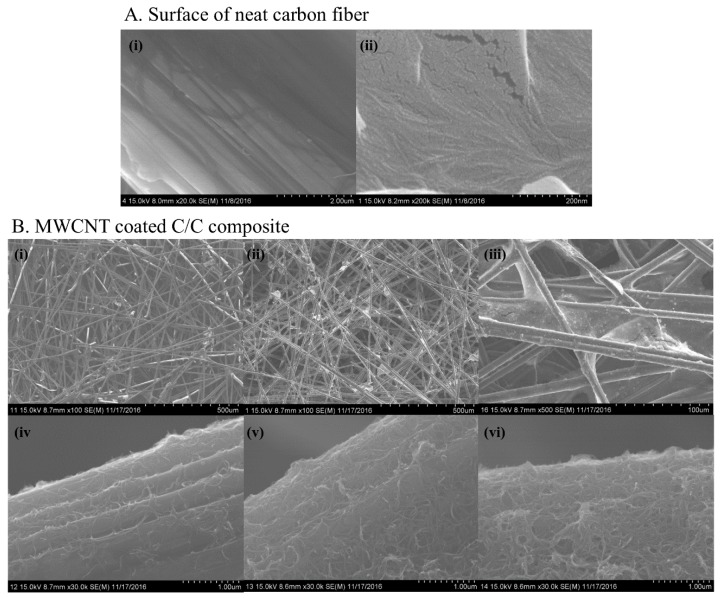
SEM images of surfaces of C/C composites: (**A**) surface of a carbon fiber in the NCFL (**i**) annular gaps (×20,000); and (**ii**) cracks (×200,000); and (**B**) (**i**) NCF; (**ii**) C-5; and (**iii**) C-10 surface of MWCNT coated C/C composites; and (**iv**)–(**vi**) the surface of a carbon fiber of C-1, C-5 and C-10, C/C composites, respectively.

The morphological features of the differently coated C/C composites and of a Nanomaterials Characterization Facility (NCF)s were characterized using SEM (scan electron microscope) images (Figure 1). The NCF is made of carbon fibers (CF) and the fibers consist of cracks and annular gaps on their surfaces (Figure 1A). These cracks and annular gaps could be filled with MWCNTs during the fabrication process of the final C/C composites (Figure 1B). With an increasing number of dipping cycles, the MWCNTs matrix connects CFs together and this decreases the porous nature of the NCF (Figure 1B(ii),(iii)). MWCNT was dispersed in water with the aid of SDS which is expected to improve MWCNT binding with the CFs [8].

For the neat carbon fiber fabric, the SEM image (Figure 1B(i)) shows a porous, smooth, and clean surface which consists of randomly packed carbon fibers. Even though the C-5 MWCNT coated C-C composite (Figure 1B(ii)) had a porous structure similar to that of a NCF (Figure 1B(i)), the carbon fibers of the carbon fabric surface were found to be homogeneously coated with MWCNTs (Figure 1B(v)). The thickness of the MWCNT layer increased with increasing the number of coating cycles, as illustrated in Figure 1B(iv)–(vi). Furthermore, in the C-10 C/C composite (Figure 1B(iii)), the porous nature of the fabric is reduced and the carbon fibers are interconnected with a continuous layer of MWCNTs. The single cycle of MWCNT coating did not cover the entire surface of the carbon fiber, as shown in Figure 1B(iv). Thus, it is evident that increasing the number of cycles will increase the covered area of the fiber and the thickness of the MWCNT layer.

#### 2.1.2. Raman Spectroscopic Analysis for Structure of Carbon Based Material

**Figure 2:**
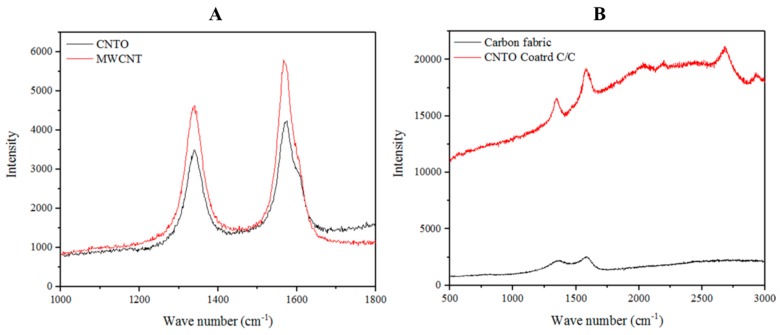
Raman spectra of MWCNT, CNTO and GN coated C/C composites: (**A**) Raman spectra of MWCNT and CNTO; and (**B**) Raman spectra of NCF and C/C composite.

Raman spectroscopy is considered as a sensitive tool for studying the structural properties of carbon materials. Raman spectra provide information about the crystalline nature of graphite-based material [22,23]. The Raman spectra of MWCNTs showed multiple peaks (Figure 2A). MWCNT had a clear D-band at 1299–1393 cm^−1^ and a distinctive G-band at 1500–1648 cm^−1^. CNTO which was obtained from oxidizing MWCNT, also displayed almost the same peaks (Figure 2A) as the ones in MWCNT. Both spectra showed the D’-band as a shoulder of the D-band at 1603 cm^−1^. This D-band is a disorder related feature arising from double resonance in the Raman scattering process. The CNTO coated C/C composite showed a Raman spectrum derived from both CNTO and Carbon fiber fabric spectra (Figure 2B) and the peak at 2540–2740 cm^−1^ is related to G’-band [23].

Carbon fibers are made of carbon based material which consists of graphite-like structure, and variations in these carbon fiber structures can be observed depending on their production method and the raw materials used [24]. NCF showed several peaks in the Raman spectrum (Figure 2B), where the 1348–1374 cm^−1^ band which is also known as the D band, could be related to the boundaries of the graphite crystals in the carbon fiber. The band at 1503–1634 cm^−1^, which is known as the G-band, arises from the vibrational energy transfer as in HOPG. Finally, the 2680–2740 cm^−1^ band in the spectrum of NCF can be related to the 2D structures in the material [22,23,24].

#### 2.1.3. X-ray Photoelectron Spectroscopy Analysis

The X-ray photoelectron spectroscopy (XPS) data were fitted using a Gaussian–Lorentzian function. For calibration purposes, the C1s electron binding energy curve was fitted to identify different environments. Different bonding environments were assigned using reported C1s chemical shifts in various organic materials [8,25] and the fitted curves are shown in Figure S1. The fabric and MWCNT contain several elements in different percentages with the higher percentage of carbon (Table S1). MWCNT was oxidized to obtain CNTO and the oxidation process increased the amount of oxygen in the MWCNT (Figure S1A,B). The number of defects as well as the C–O bonds increased due to the oxidation. However, the increase of C=O bonds is not clear, which may be due to the mild oxidation process occurred by NH_4_OH and H_2_O_2_ that resulted in a higher amount of –OH groups than –COOH groups [25].

Carbon fabric itself contains 8.8% Oxygen. However, C1s peaks analogous to C–O or C=O bonds were not recognizable in the C1s curve of the NCF (Figure S1A). In the XPS graph of the MWCNT coated sample (Figure S1B), peaks corresponding to bonds at 286.5 eV and 286.8 eV could be observed and this might be due to the CNTO coating on the fabric. Na and S present in the MWCNT coated NCF originates from SDS used in the CNTO suspension (Table S1).

### 2.2. Tensile Strength of MWCNT Coated C/C Composites

Tensile strength is an important factor regarding the usability of EMI shielding materials such as polymer composites, shielding textiles, carbon fabrics, etc. Even if carbon fibers (CFs) are considered as fine materials used to improve the tensile strength of carbon composites due to their high stiffness, strength, and lightness, the tensile strength of a carbon fabric made of the CFs depends upon the thickness and arrangement of the CFs in the fabric. Since a neat carbon fiber fabric consists of a randomly deposited fiber matrix, the interfacial bonding between CFs is considerably weak, which results in low tensile strength of the NCF [26,27].

MWCNTs are perfect candidates for nanoscale reinforcement of carbon fabric due to their outstanding mechanical, electrical, and thermal properties [26]. The high interfacial binding strength between MWCNTs and CFs results carbon composites with much higher tensile strength compared to neat carbon fabric [27]. The MWCNT coated C/C composites showed a considerable increase in tensile strength compared to the neat carbon fiber fabric, as listed in Table S2. The C/C composites coated with 1 g L^−1^ MWCNT showed >1.5-fold increase in the tensile strength while the composites coated with 2 g L^−1^ MWCNT showed more than a three-fold increase. This may be due to coating each single CF surface with MWCNT which leads to interconnect them together by a MWCNT matrix as was observed in SEM images (Figure S2).

In all tensile strength curves illustrated in Figure S2, the linear region (the elastic region) of the graphs is very short. This is due to the inherent low tensile capability of the neat carbon fabric. Since NCF consists of a randomly deposited carbon fiber matrix, it tends to break even at a very small external force and does not show a significant elastic region in the tensile stress curve. However, coating increases the tensile strength and elastic region of the carbon composites significantly (Figure S2 and Table S2).

### 2.3. Electromagnetic Shielding Effectiveness of MWCNT Coated Carbon Fiber Fabric

**Figure 3:**
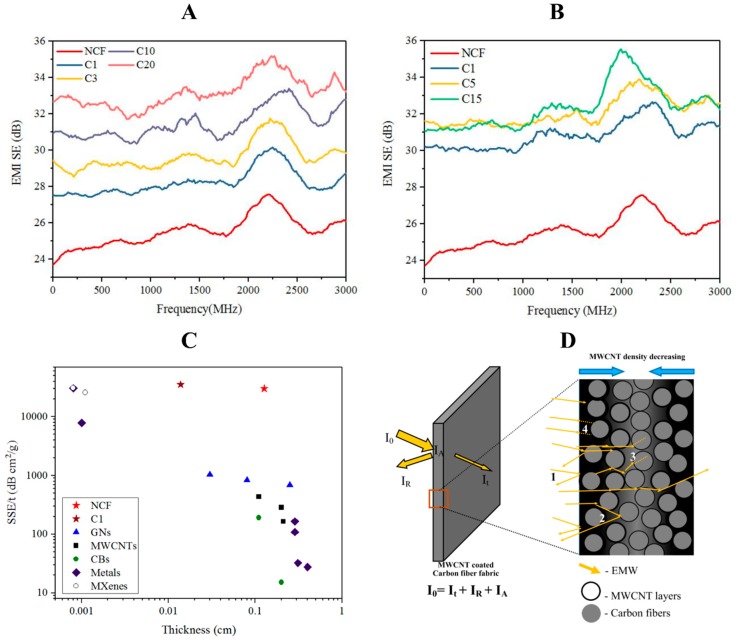
The EMI shielding effectiveness of MWCNT coated carbon fabric and the NCF: (**A**) EMI SE of 1 g L^−1^ CNTO coated fabrics; (**B**) EMI SE of 2 g L^−1^ CNTO coated fabrics; (**C**) absolute EMI shielding (SSE/t) vs. thickness of the material of MWCNT coated C/C composites from current work with previously reported shielding composites from the data recorded in Table S4; and (**D**) proposed EMI shielding mechanism of the MWCNT coated C/C composites. (1) Immediately reflected EMWs; (2) EMWs reflected by internal layers of MWCNT/ carbon fibers; (3) absorbed EMWs released as heat causing Ohmic loss by MWCNTs; and (4) absorbed EMWs released as heat causing Ohmic loss by Carbon fibers. I_0_ = I_t_ + I_R_ + I_A_ where, I_0_ is the intensity of EMW, I_t_ is transmitted intensity, I_R_ is reflected intensity and I_A_ is absorbed intensity.

NFCs were coated with MWCNTs in SDS dissolved in water. The fabrics were dip coated and the number of coating cycles was the method of maintaining the thickness of the coating. EMI-SE also increased with the number of cycles (Figure 3). MWCNT 1 g L^−1^ coating showed a methodical increase in EMI-SE whereas the 2 g L^−1^ MWCNT coating did not display a methodical increase. Therefore, a 1 g L^−1^ solution would be more useful in a multiple-cycles coating process. However, single dip coating of 2 g L^−1^ increased the EMI-SE significantly compared to 1 g L^−1^ solution based dip coating (Figure 3). The composites show significant increase in EMI-SE in the 1900–2500 MHz frequency range which consists of Wi-Fi and other radio signals used in mobile devices [28]. Therefore, the MWCNT coated C/C composites would be an ideal to produce a shield for those frequencies.

Two layers of the fabric showed a higher EMI-SE compared to one layer fabric where a single layer had a thickness ~150 µm and two of them had ~300–350 µm. The highest shielding effectiveness counts observed were 52.5 dB, 55.9 dB, 68.3 dB and 61.5 dB at 840 MHz, 2295 MHz, 2700 MHz, and 2850 MHz respectively (Figure S3). C_1_ with 2 g L^−1^ coating showed the highest increase in EMI-SE (~30 dB) (Figure 3). The increase in EMI-SE of C_1_ composite with 1 g L^−1^ MWCNT coating was limited to ~27 dB. Furthermore, the composites fabricated in the current study displayed extensively higher absolute EMI SE values (SSE/t) compared to previously reported EMI SE materials (Figure 3C). C_1_ composites in 1 g L^−1^ showed the highest SSE/t value, ~35,000 dB cm^2^ g^−1^. However, NCF showed a moderately high SSE/t value as well. The SSE/t values decreased when the number of coating cycles were increased. This is due to the increase in the density of the material (Table S3). Considering the previously reported carbon based materials, C_1_ shows the highest absolute EMI shielding effectiveness.

The EMI SE of the MWCNT coated C/C composites can be described using several proposed mechanisms. The mechanisms can be explained in a few steps: MWCNTs consist of graphitic structures which was confirmed using Raman spectra (Figure 3D). As electromagnetic waves (EMW) hit the surface of the composite, some of the EMWs are immediately reflected due to the abundant free electrons on the surface of Graphene or MWCNTs [8]. Some of the remaining EMWs pass through the CFs and MWCNT based or CFs based matrices where they interact with the high density of electron clouds in the matrices and induce a current causing Ohmic losses [29]. The remaining EMWs, after passing through the first layer of MWCNT, encounter the next barrier and the phenomenon of EMW attenuation is repeated. Simultaneously the inner layer acts as a reflecting surface and gives rise to multiple reflections resulting in further reduction of the total transmission of EMWs.

### 2.4. Thermal Stability and Thermogravimetric Analysis of the C/C Composites

The thermal stability of nanocomposites was studied using simultaneous measurements of DSC and thermogravimetric analysis (TGA) methods. The mass loss from TGA and the enthalpy changes from DSC were compared in a temperature range spreading from room temperature to 1000 °C to identify the specific mechanisms of composite degradation under N_2_ atmosphere at a heating rate of 10 °C min^−1^.

**Figure 4:**
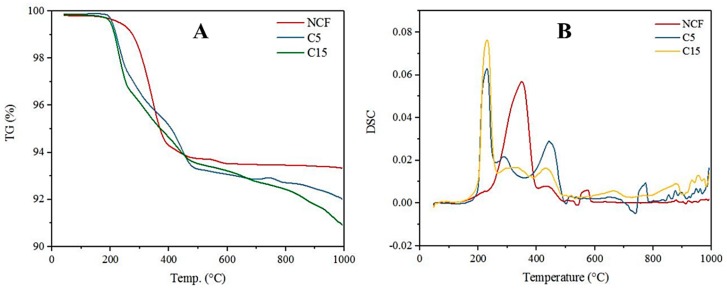
TGA-DSC curves of C/C composites: (**A**) TGA curves of MWCNT coated C/C composites and NCF; and (**B**) DSC curves of MWCNT coated C/C composites and NCF.

In an oxygen-free atmosphere, degradation of the composites was inhibited up to 284 °C. In contrast, composite degradation occurred rapidly from 300 to 400 °C. All the composites showed less than 10% weight loss up to 1000 °C (Figure 4A). In the DSC curve depicting the neat carbon fabric, an endothermic peak appeared near 356 °C which can be associated with the decomposition of C/C composite [30]. This peak shifted to 223–228 °C and the intensity of the peak increased dramatically in MWCNT coated carbon C/C composites [31]. This may be due to the introduction of SDS and MWCNT in to the NCF. However, MWCNT coated C/C composites exhibit outstanding stability over higher temperatures (Figure 4). Even though overall stability of the samples is relatively high, it is lower than the NCF stability. This might be due to the loss of loosely bound MWCNTs from the surface of the C/C composites.

### 2.5. Electrical Conductivity 

Well dispersed CNTs possess higher electrical conductivity and EMI shielding due to its higher aspect ratio [6,8,20]. Electrical conductivity of the MWCNT coated C/C composites were fabricated with different dip-coating cycles. The coating process significantly affected the electrical conductivity of NCF.

The fabricated C/C composites showed higher electrical conductivity in the range of 11.02–16.42 S cm^−1^. NCF made of carbon nanofibers showed 16.32 S cm^−1^ electrical conductivity (Table S5). MWCNT coating showed a reduction in the conductivity in single coating. The conductivity of the C/C composites increased with the number of coatings to the maximum of 15.65 S cm^−1^ in the C_15_ C/C composite that was fabricated using 1 g L^−1^ MWCNT coatings. The reduction of the conductivity occurred probably due to the interface resistance and the resistance due to the elevation of the thickness [32,33]. Conductivity of MWCNT might be slightly reduced with the oxidation process. Earlier studies showed significantly low conductivity in composites with a low amount of CNT loadings. Du et al. (2005), reported that the carbon nanotube/polymer composites with enhanced electrical conductivity with 3% addition of single walled carbon nanotubes (SWCNT) showed 4.2 × 10^−3^ S cm^−1^ [34] and Kim et al. (2005) reported MWCNT epoxy composites with a conductivity of 4.8 × 10^−3^ S cm^−1^ [32]. However, Arjmand et al. (2016) achieved 16.67 S cm^−1^ conductivity in the compressed powders of N-doped CNTs. The increased conductivity of the N-doped CNT is due to the enhanced electron density of N atoms [33]. The current study reported the fabrication of a free standing film made of MWCNT and CFs where the core of the material was CFs. This material reached an electrical conductivity of 11.02–16.42 S cm^−1^ without doping of metals or nitrogen.

## 3. Materials and Methods

### 3.1. Materials

Wet-laid non-woven carbon fabric (NCF) (basis weight of 20.2 g/m^3^, thickness of 190 µm and basis weight of 19.2 g/m^2^, thickness of 150 µm) was purchased from Clean & Science Co. Ltd. (Seoul, Korea). MWCNTs (CM-90, 90 wt % diameter of 20 nm and length of 100 µm) was purchased from Applied Carbon Technology Co. Ltd. (Pohang, Korea). Sodium dodecyl Sulfate (SDS) 98%, Ammonia solution 28 wt % in water was purchased from Sigma Aldrich (St. Louis, MO, USA). Hydrogen peroxide 34.6 wt % was from Samchun Pure Chemicals (Pyumgtack, Korea). All the chemicals were used without any further purification.

### 3.2. Oxidation of Multi-Walled Carbon Nanotubes

A portion of 1.0 g of the MWCNTs was dispersed in 80 mL of the mixture of ammonium hydroxide (28 wt %) and hydrogen peroxide (34.6 wt %) in a 1:1 ratio in a 250-mL round bottom flask equipped with a condenser for 15 min at room temperature. The dispersion was heated to 80 °C and kept for 5 h. The resulting dispersion was diluted in water and filtered. Then the resulting solid (MWCNT oxide) was washed with deionized water until the neutral pH was obtained. Finally, the sample was dried in a vacuum at 40 °C overnight. The resulting solid is MWCNT oxide (CNTO).

### 3.3. Preparation of MWCNT Oxide Dispersion

An amount of 0.25 g of CNTO and 0.25 g of SDS were dispersed in 250 mL of water using ultra sonication (UIL MINI Ultrasonic, UIL Ultrasonic Co., Ltd., Gyeonggi-do, Korea) for 15 min and the dispersion was refluxed at ~190 °C for 5 h. The refluxed dispersion was transferred to a flask and sonicated for another 3 h to obtain a 1 g L^−1^ CNTO solution. A solution of 2 g L^−1^ CNTO was prepared using the previously mentioned method except for employing initial amounts of 0.5 g CNTO and 0.5 g SDS in 250 mL of deionized water.

### 3.4. Preparation of GN and CNT Coated C/C Composite

**Figure 5:**
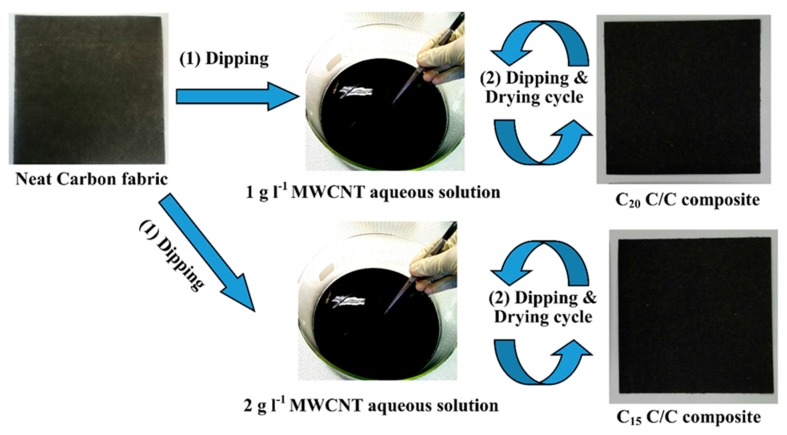
Schematic procedure to manufacture C/C composites by dip-coating technique.

A series of MWCNT/carbon fabrics were manufactured using a dip-coating process, as shown schematically in Figure 1. A neat carbon fabric (19.2 g m^−2^) was dipped into the previously made 1 g L^−1^ or 2 g L^−1^ MWCNT dispersion accordingly, at room temperature and was dried at >100 °C for 5 min. This dip coating process was repeated by 20 cycles to adjust the amount of MWCNT coated on the carbon fabric. The final MWCNT coated carbon fabrics were named as C-x, where x stands for the cycle number of the dip-coating process as shown in Figure 5.

### 3.5. Characterization

The dispersion state of each coating and the associated morphology was characterized by obtaining the surface and cross section images with the aid of a field emission scan electron microscope (SEM, S-4800, Hitachi, Japan).

The structural features of pristine MWCNT, CNTO, NCF and C/C composites were identified using a high-resolution Raman spectrophotometer with an excitation wavelength: 532 nm, laser spot size: 5 µm, exposure time: 30 s. (Renishaw RM1000 inVia, Vancouver, BC, Canada). The percentage of elements and the chemical environment of carbon materials were characterized using XPS analysis with an Al anode and the spot size: 30–400 µm at 100 W of E_max_ (K-Alpha, Thermo Fisher, East Grinstead, UK).

The tensile strength of the coated samples was determined using fabric samples with dimensions of 100 mm × 50 mm with the aid of an Extensometer at 2000 N, 1.3 mm/min (Instron 556A-AVE 2, Norwood, MA, USA). The electrical properties were investigated employing the four-probe method using C/C composites and NCF disks with a 30-mm diameter (FPP-RS8, DASOL ENG, Seoul, Korea). Thermogravimetric data were measured using Thermal analyzer (DSC TMA Q400, TA Instruments Ltd., New Castle, DE, USA).

The EMI shielding effectiveness (SE) of the C/C composites and neat carbon fabrics was measured at room temperature in accordance with a shielding effectiveness measurement system under an EMI shielding tent (ASTM-D4935-10, ASTM International, West Kentucky, PA, USA). The resulted data were plotted and smoothed using the Savitzky–Golay function (Origin 2017 graphing and analysis, OriginLab (Boston, MA, USA).

## 4. Conclusions

A series of MWCNT coated C/C composites with a high flexibility, low apparent density (~0.066–0.1 g cm^−3^) and low thickness (0.12–0.20 mm) were successfully fabricated using a facile dip coating process. SEM images of the surface showed that the composite consists of nanopores and MWCNTs were deposited on the surface of the carbon fibers of the fabric as well as in the inter fiber spaces. In an oxygen-free atmosphere, the thermal degradation of the composites was strongly inhibited up to the temperature of 284 °C, and the degradation initiated rapidly from 300 to 400 °C. All the composites showed less than 10% weight loss up to 1000 °C. The electrical conductivity increased in 2 g L^−1^ MWCNT coated C_5_. The tensile strength of the composites increased significantly due to the coating process. NCF showed 11.21 kgf cm^−2^, whereas the C_1_ and C_10_ C/C composites (1 g L^−1^) showed 17.39 kgf cm^−2^ and 68.28 kgf cm^−2^, respectively. The fabricated composites had a high EMI shielding effectiveness, whereas the MWCNT coated composites showed 33 dB with the highest EMI-SE of 38 dB. Two layers of C/C composites gave the highest SMI SE of 68 dB at 2.7 GHz. Moreover, MWCNT coated composites showed ~35,000 dB cm^2^ g^−1^ absolute and 486.54 dB cm^3^ g^−1^ specific EMI SE. In contrast, the C/C composites that were obtained by the facile coating process of MWCNT had higher EMI SE, electrical conductivity at a lesser thickness and a lower density with better flexibility. These composites thus exhibit high prospective in advanced application areas such as aerospace and auto mobile applications.

## Supplementary Materials

The following are available online at www.mdpi.com/1996-1944/10/12/1350/s1, Figure S1: XPS graphs of neat carbon fabric, Figure S2: Tensile strains curves, Figure S3: EMI-SE, Figure S4: XRD patterns of curve, Figure S5: Optical image of free standing films, Table S1: XPS results, Table S2: Maximum stencil strength, Table S3: Specific EMI shielding effectiveness and absolute effectiveness of MWCNT coated C/C composites, Table S4: Specific EMI shielding effectiveness and absolute effectiveness of various solid materials, Table S5: Thermal, electrical conductivity and specific shielding effectiveness.
