# Supplementary material for: MWCNT Coated Free-Standing Carbon Fiber Fabric for Enhanced Performance in EMI Shielding with a Higher Absolute EMI SE
Source: Materials (Basel). 2017 Nov 24;10(12):1350. doi: 10.3390/ma10121350 (PMC5744285; doi:10.3390/ma10121350)
Supplement: Supplementary file 1 [file materials-10-01350-s001.pdf]

materials

MDPI

Supplementary Materials

MWCNT coated free-standing carbon fiber fabric for enhanced performance in EMI shielding with a higher absolute EMI SE

Supplementary Texts

Electrical conductivity

The electrical conductivity increased while the sheet resistance decreased with the coating process. The resistance of a material can be calculated as [1],

$$R = \rho \frac{L}{A} = \rho \frac{L}{Wt}$$

where, R is the material resistance, ρ is the resistivity, A is the cross-sectional area, and L is the length. The cross-sectional area can be separated into the width (W) and the sheet thickness (t).

$$R = \frac{\rho}{t}\frac{L}{W} = R_s \frac{L}{W}$$ R_s - Sheet resistance

If the film thickness (t) is known, t and R_s can be multiplied to obtain the bulk resistivity ρ (in Ω cm):

$$\rho = R_s . t$$

The reciprocal of the resistivity is the conductivity of the material. $\sigma = \frac{1}{\rho}$ where, the conductivity of the material can be give as,

$$\sigma = (R_s . t)^{-1}$$

The conductivity of the material was calculated according to the above equation.

Electromagnetic Interference (EMI) Shielding Measurements

The electromagnetic interference shielding effectiveness (EMI SE), is a measure of blocking electromagnetic waves (EMW).

EMI SE is experimentally defined as the logarithmic ratio of incoming power (P_I) to transmitted power (P_T) [2] which is measured in decibel (dB),

$$\text{SE (dB)} = \log_{10}\left(\frac{P_I}{P_T}\right)$$

When an EM radiation is incident on shielding film, the reflected power (P_R), absorbed power (P_A), and transmitted power (P_T) must add up to incident power (P_I), that is,

$$P_I = P_R + P_A + P_T$$

For the intensity (*I*) it is,

$$I_0 = I_R + I_A + I_T$$

Specific Shielding Effectiveness (SSE)

Mathematically, SSE is calculated dividing the EMI SE by the density of material (ϱ) [3].

SSE = EMI SE/density (dB cm^3 g^{-1})

SSE gives a more accurate account on EMI SE considering ϱ of the material where, some light weight material might be having higher EMI SE.

SSE does not account for the thickness information of the material. A material with a large thickness may result in a higher SSE value while maintaining a low density. The following equation is used to evaluate the absolute effectiveness (SSE/t) of a material in relation to the thickness [3–5].

$$SSEt = SSE/t \ (\text{dB cm3 g}^{-1}\ \text{cm}^{-1} = \text{dB cm}^{2}\ \text{g}^{-1})$$

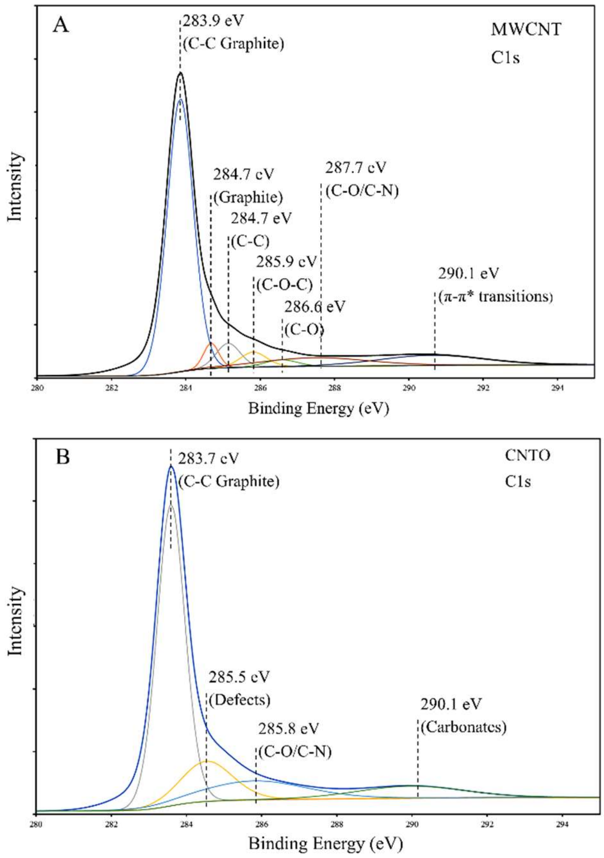

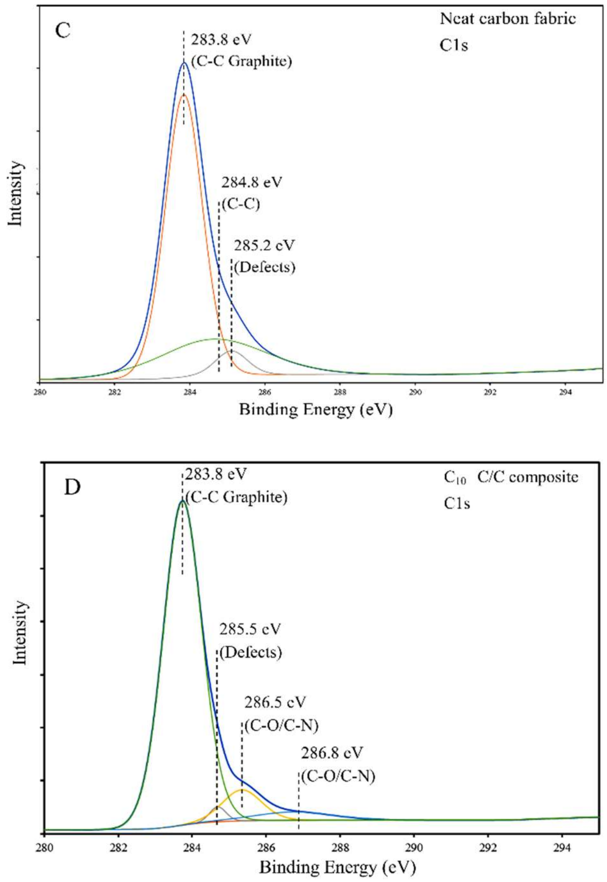

Figure S1. XPS graphs of neat carbon fabric (A) XPS C1s peaks of MWCNT coated carbon fabric (B) XPS C1s peaks of CNTO (C) XPS C1s peaks of neat carbon fabric (D) XPS C1s peaks of MWCNT coated C/C composite.

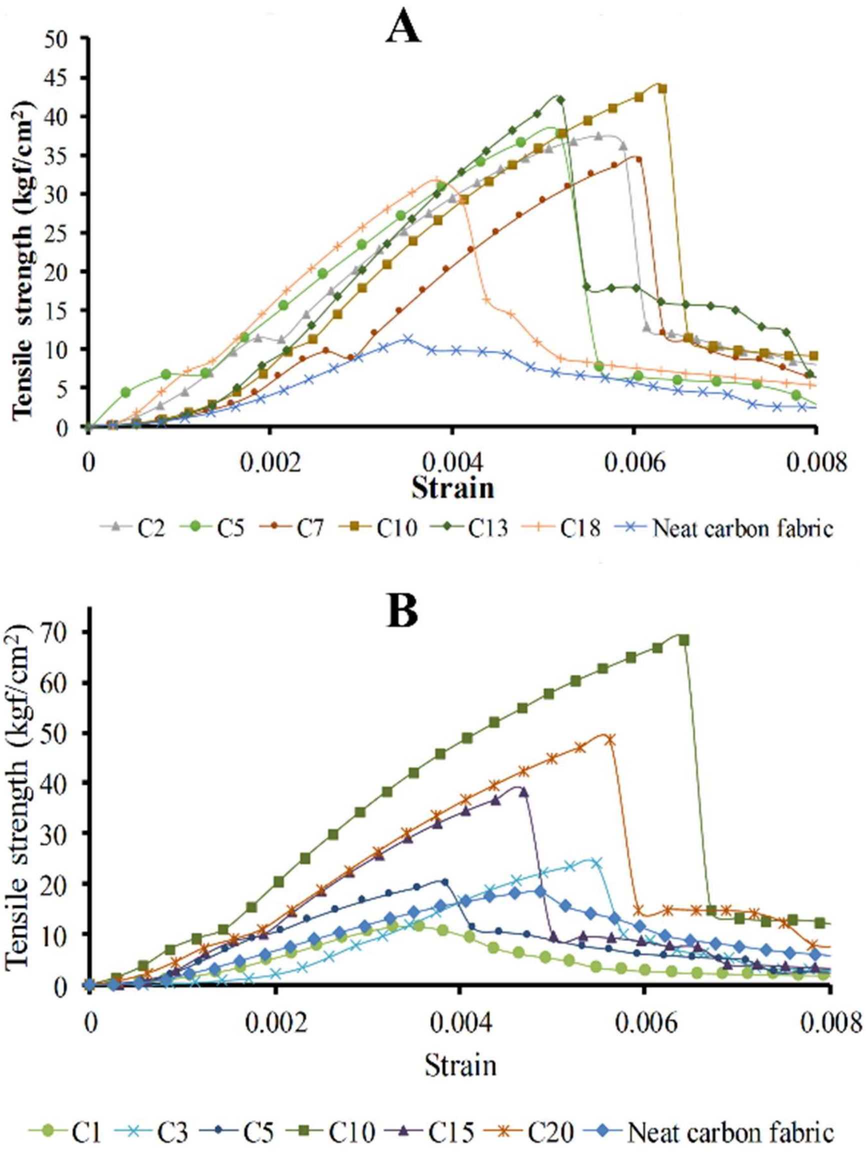

Figure S2. Tensile-strain curves of MWCNT and GN coated C/C composites. (A) Tensile-strain curves of 1 g/l MWCNT coated C/C composites. (B) Tensile-strain curves of 2 g/l MWCNT coated C/C composites.

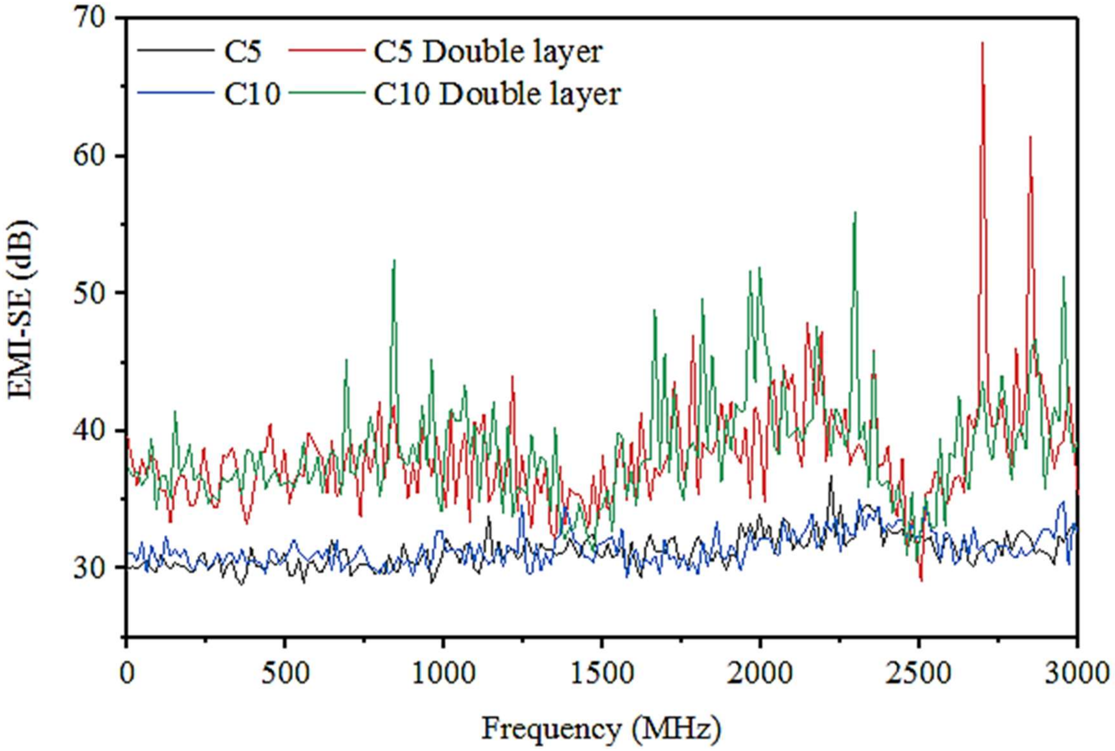

Figure S3. EMI SE of 1 g/l MWCNT coated C/C composites and respective single layers.

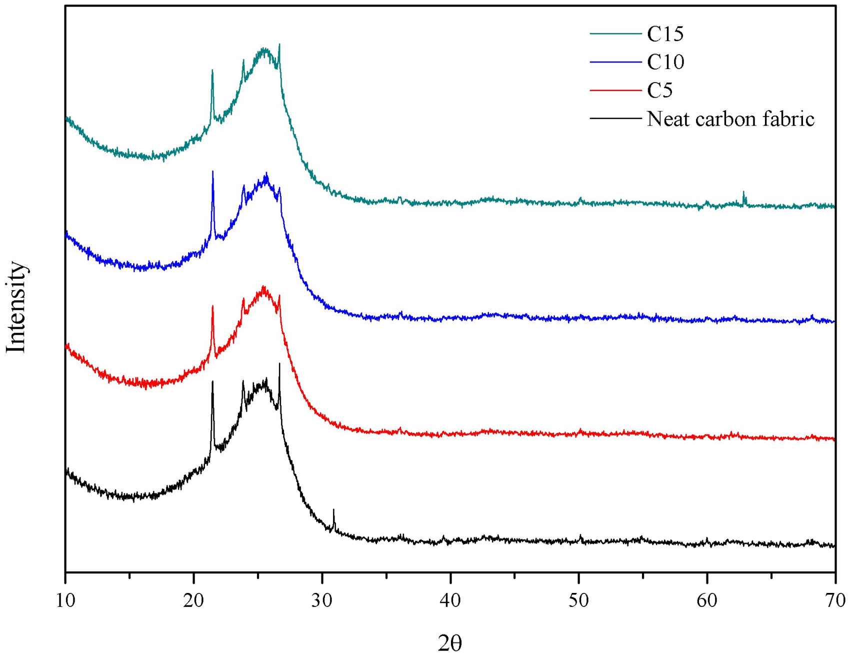

Figure S4. XRD patterns of curves of MWCNT coated C/C composites.

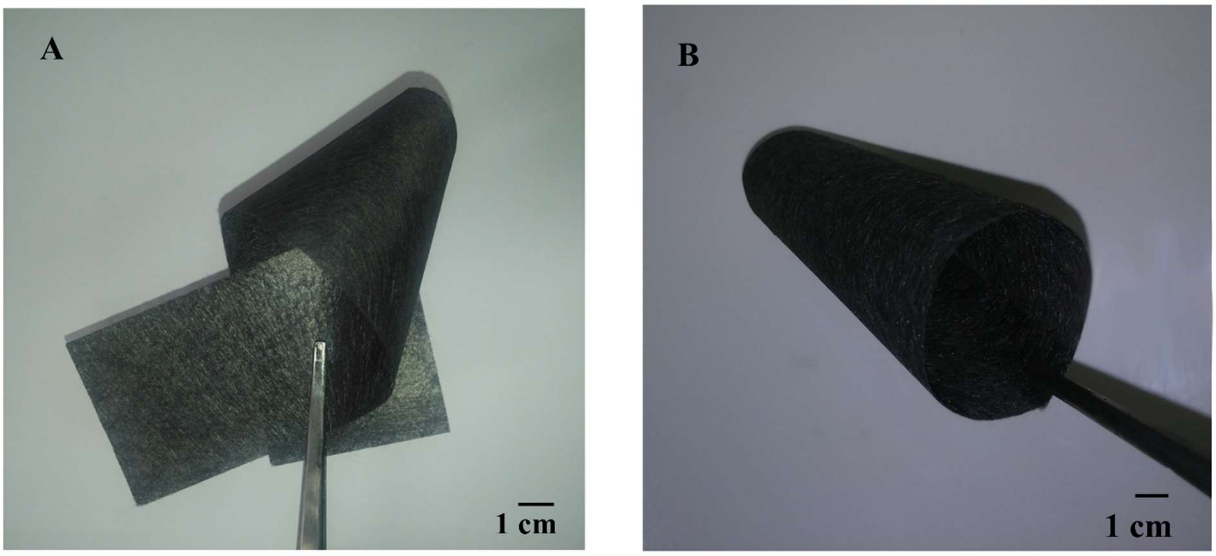

Figure S5. Optical images of free-standing films. Digital photographs of (A) Neat carbon fabric as received. (B) MWCNT coated C/C composites (C_1). Films fabricated were flexible and not transparent.

Table S1. XPS results for MWCNTs, CNTOs, neat carbon fabric and MWCNT (1 g/l) coated samples.

	**MWCNT**	**CNTO**	**Fabric**	**C_5**	**C_{10}**	**C_{15}**	**C_{20}**
Element				At. %			
C1s	97.38	91.3	84.23	84.49	84.08	89.53	86.28
O1s	2.19	5.91	8.8	11.72	11.54	7.55	8.89
S2p	0.43	1.59		2.16	2.5	1.52	1.97
Na1s				1.63	1.88	1.4	1.75
N1s		1.2	1.16				1.1
Si2p			0.51				

Table S2. Maximum tensile strengths of CNTO coated C/C composites.

**CNT (2 g/l)**	**coating cycle**	**Neat fabric**	**2**	**5**	**7**	**10**	**13**	**18**
	Tensile strength (kgf/cm^2)	11.21	37.37	38.29	52.69	43.48	48.00	67.05
	Fold-vice increase		3.3	3.4	4.7	3.2	4.3	6.0
CNT (1 g/l)	coating cycle		1	3	5	10	15	20
	tensile strength (kgf/cm^2)		17.39	24.09	20.13	68.28	50.31	48.51
	Fold-vice increase		1.6	2.1	1.8	6.1	4.5	4.3

Table S3. Specific EMI shielding effectiveness (SSE) and absolute effectiveness (SSE/t) of MWCNT coated C/C composites.

MWCNT concentration	Name	ϱ (g cm^{-3})	Ave. SE (dB)	SSE (dB cm^{3}g^{-1})	SSE/t (dB cm^2 g^{-1})
**1 g/L**	C_1	0.058	28.21	486.54	35256.75
	C_3	0.069	29.66	429.97	28856.96
	C_5	0.071	31.32	441.02	26251.43
	C_{10}	0.077	31.47	408.74	23223.86
	C_{15}	0.076	31.54	415.05	25154.77
	C_{20}	0.080	33.20	415.12	21072.17
**2 g/L**	C_1	0.083	30.87	371.97	20550.75
	C_5	0.081	32.12	396.63	21792.73
	C_{15}	0.102	32.40	317.72	18052.40
	NCF	0.067	25.56	381.50	30039.42

Table S4. Specific EMI shielding effectiveness (SSE) and absolute effectiveness (SSEt) various solid structure materials.

Type	Filling material	Filler (Wt %)	Polymer matrix	t (cm)	SE (dB)	SSE (dB cm^3g^{-1})	SSE/t (dB $cm^2 g^{-1}$)	Ref
Carbon based	GN	7		0.25	45.1	173	692	[6]
	GN	25	PEDOT	0.08	70	67.3	841	[7]
	GN/Fe3O4	Bulk	*	0.03	24	31	1033	[8]
	MWCNT	15	ABS	0.11	50	47.6	432.7	[9]
	MWCNT	20	PC	0.21	39	34.5	154	[10]
	MWCNT	20	PS	0.2	30	57	285	[11]
	CB	15	ABS	0.11	20	20.9	190	[9]
	CB	37.5	EPDM	0.2	18	30.3	15.1	[12]
	CNT	*	Polymeric	0.35	80	*	*	[14]
Metal based	Cu	Bulk	*	0.31	90	10	32.3	[13]
	Ni fiber	7	PES	0.285	58	31	108.7	[13]
	Ni filaments	7	PES	0.285	87	47	164.9	[13]
	Stainless steel	Bulk	*	0.4	89	11	27.5	[13]
	Cu foil	Bulk	*	0.0010	70	7.8	7812	[5]
	Al foil	Bulk	*	0.0008	66	24.4	30555	[5]
CF & MWCNT	†NCF	CF bulk	*	0.0127	25.56	381.50	30039	This work
	†C_1	CF/CNT	*	0.0138	28.22	486.54	35256	

* Sign indicates that the values were impossible to calculate or not available enough data to calculate. † Densities of NCF and C_1 were 0.067 g cm^{-3} and 0.058 g cm^{-3} respectively. C_1 and NCF show better specific EMI shielding effectiveness compared to other reported materials. Foams are used in different purposes where the thickness is relatively higher compared to solid structured films.

Table S5. Thermal conductivity and Electrical conductivity and specific shielding effectiveness of MWCNT coated C/C composites.

C of coating solution	Name	R_s (Ω/sq)	σ S/cm	SSE dB cm^3 g^{-1}
1 g/l	C_1	5.465	13.259	486.54
	C_3	5.852	11.467	429.97
	C_5	4.803	12.393	441.02
	C_{10}	4.078	13.930	408.74
	C_{15}	3.871	15.655	415.05
2 g/l	C_1	5.013	11.020	371.97
	C_5	3.345	16.424	396.63
	C_{15}	3.506	16.205	317.72
	NCF	4.823	16.325	381.50

NCF- Neat carbon fabric

References

1. Han, Z.; A. Fina, Thermal conductivity of carbon nanotubes and their polymer nanocomposites: A review. *Prog. Polym. Sci.* **2011**, 36, 914–944.

2. Bian, X.M.; Liu, L.; Li, H.B.; Wang, C.Y.; Xie, Q.; Zhao, Q.L.; Bi, S.; Hou, Z.L.; Construction of three-dimensional graphene interfaces into carbon fiber textiles for increasing deposition of nickel nanoparticles: flexible hierarchical magnetic textile composites for strong electromagnetic shielding. *Nanotechnology* **2017**, 28, 45710.
3. Zeng, Z.; Jin, H.; Chen, M.; Li, W.; Zhou, L.; Zhang, Z. Lightweight and Anisotropic Porous MWCNT/WPU Composites for Ultrahigh Performance Electromagnetic Interference Shielding. *Adv. Funct. Mater.* **2016**, *26*, 303–310.
4. Ameli, A.; Nofar, M.; Wang, S.; Park, C.B. Lightweight Polypropylene / Stainless-Steel Fiber Composite Foams with Low Percolation for E ffi cient Electromagnetic Interference Shielding. *Appl. Mater. Interfaces.* **2014**, *6*, 11091–11100.
5. Shahzad, F.; Alhabeb, M.; Hatter, C.B.; Anasori, B.; Hong, S.M.; Koo, C.M.; Gogotsi, Y. Electromagnetic interference shielding with 2D transition metal carbides (MXenes). *Science* **2016**, *353*, 1137–1140.
6. Yan, D.X.; Pang, H.; Li, B.; Vajtai, R.; Xu, L.; Ren, P.G; Wang, J.H.; Li, Z.M.; Structured reduced graphene oxide/polymer composites for ultra-efficient electromagnetic interference shielding. *Adv. Funct. Mater.* **2015**, *25*, 559–566.
7. Agnihotri, N.; Chakrabarti, K.; De, A.; Highly efficient electromagnetic interference shielding using graphite nanoplatelet/poly(3,4-ethylenedioxythiophene)-poly(styrenesulfonate) composites with enhanced thermal conductivity. *RSC Adv.* **2015**, *5*, 43765–43771.
8. Song, W.L.; Guan, X.T.; Fan, L.Z.; Cao, W.Q.; Wang, C.Y.; Zhao, Q.L.; Cao, M.S. Magnetic and conductive graphene papers toward thin layers of effective electromagnetic shielding. *J. Mater. Chem. A.* **2015**, *3*, 2097–2107.
9. Al-Saleh, M.H.; Saadeh, W.H.; Sundararaj, U. EMI shielding effectiveness of carbon based nanostructured polymeric materials: A comparative study. *Carbon N. Y.* **2013**, *60*, 146–156.
10. Pande, S.; Chaudhary, A.; Patel, D.; Singh, B.P.; Mathur, R.B. Mechanical and electrical properties of multiwall carbon nanotube/polycarbonate composites for electrostatic discharge and electromagnetic interference shielding applications. *RSC Adv.* **2014**, *4*, 13839
11. Arjmand, M.; Apperley, T.; Okoniewski, M.; Sundararaj, U. Comparative study of electromagnetic interference shielding properties of injection molded versus compression molded multi-walled carbon nanotube/polystyrene composites. *Carbon N. Y.* **2012**, *50*, 5126–5134.
12. Ghosh, P.; Chakrabarti, A. Conducting carbon black filled EVA vulcanizates: Assessment of dependence of physical and mechanical properties and conducting character on variation of filler loading. *J. Polym. Mater.* **2000**, *17*, 291–304.
13. Shui, X.; Chung, D.D.L. Nickel filament polymer-matrix composites with low surface impedance and high electromagnetic interference shielding effectiveness. *J. Electron. Mater.* **1997**, *26*, 928–934.
14. Micheli, D.; Vricella, A.; Pastore, R.; Delfini, A.; Giusti, A.; Albano, M.; Primiani, V. M. Ballistic and electromagnetic shielding behaviour of multifunctional Kevlar fiber reinforced epoxy composites modified by carbon nanotubes. *Carbon* **2016**, *104*, 141-156.

© 2017 by the authors. Submitted for possible open access publication under the terms and conditions of the Creative Commons Attribution (CC BY) license (http://creativecommons.org/licenses/by/4.0/).
